# Topology-Consistent Trajectory Event Matching for GNSS-Denied Vehicle Initial Localization

**DOI:** 10.3390/biomimetics11100761

**Published:** 2026-10-21

**Authors:** Haoxin Cai, Minghong Zhu, Weiqi Xu, Ming Jin, Chen Hua, Zhenpeng Wang

**Affiliations:** 1 Faculty of Electrical Engineering and Computer Science, Ningbo University, Ningbo 315211, China; 2 System Engineering Institute of Sichuan Aerospace, Chengdu 610100, China

**Keywords:** GNSS-denied positioning, vehicle initial localization, trajectory event matching, topological consistency, bio-inspired navigation, road-network matching

## Abstract

Vehicle initial localization under global navigation satellite system (GNSS)-denied conditions remains challenging because an odometry trajectory provides relative motion but no absolute road-network reference. Inspired by the coordinated use of self-motion information, recognizable route cues, and route continuity in biological navigation, this paper proposes a topology-consistent trajectory-event matching method. The method organizes odometry into ordered trajectory structure pairs formed by adjacent normal turning events and their intervening corridors, while a standard road-network map is automatically enhanced with internal direction-change points to construct corresponding map-side structures. Local candidates are retrieved according to direction, length, turning, and corridor-morphology consistency, and successive candidates are jointly confirmed under topological continuity. The confirmed discrete correspondence is then refined by local trajectory-image matching to estimate the continuous starting position. Experiments on KITTI-360 sequences 00, 08, and 09 with 135 starting positions yield an overall initialization success rate of 88.9%; successful trials achieve a mean error of 2.46 m and a median error of 2.29 m. The results show consistent performance across the evaluated road scenarios while indicating that sparse turning events and locally ambiguous road structures remain important limiting conditions.

## 1. Introduction

Global navigation satellite systems (GNSS) are the primary source of absolute positioning information for vehicles. However, in urban canyons, tunnels, underground roads, tree-lined streets, and environments with electromagnetic interference, GNSS signals are prone to degradation or interruption. In such cases, odometry can provide continuous short-term relative motion information, but the resulting trajectory is expressed in a local coordinate frame and cannot directly determine the absolute starting position of the vehicle in a road network. Therefore, estimating the initial vehicle position using only the odometry trajectory during the initialization stage and a regional road-network map is a key problem for recovering the map reference under GNSS-denied conditions.

Existing studies have improved localization reliability in GNSS-challenged environments by using vision, light detection and ranging (LiDAR), high-definition maps, semantic landmarks, or multisensor fusion [1,2]. Representative visual-inertial and LiDAR-inertial systems illustrate the accuracy achievable through tightly coupled state estimation and mapping [3,4,5,6], while visual place recognition provides another means of associating observations with known environments [7]. These methods can achieve high accuracy, but they usually rely on additional sensors, pre-built environmental models, or external features that must be stably observable. In contrast, standard road-network maps are widely available and inexpensive to obtain, while providing relatively stable geometric and topological information that can serve as environmental references for low-cost initial localization.

Under such an information setting, GNSS-denied localization bears a functional resemblance to biological navigation in the absence of global position information. Desert ants provide a representative example: they use path integration to maintain direction and distance information from self-motion, while visual landmarks and learned route segments support navigation along familiar routes [8,9,10]. Studies of ant navigation further show how path integration, familiar visual scenes, and route experience provide complementary directional information [11,12,13,14]. Recent field experiments indicate that changes in familiar visual scenes alter route-following and scanning behavior, underscoring the role of learned visual route cues [15]. For GNSS-denied vehicles, odometry similarly preserves motion information, whereas the road network provides geometric, directional, connectivity, and topological cues. This resemblance motivates a structure-aware localization strategy that combines trajectory motion structures with road-network relations and uses consistency across successive structures to reduce local ambiguity.

Road-network-map-based localization methods have shown that road shape, turning relationships, and connectivity constraints can provide useful references for vehicle trajectories [16,17,18,19]. Conventional map matching associates noisy geographic observations with road segments using geometric and topological rules or probabilistic models [20,21]. For sparsely sampled trajectories, candidate paths and temporal or sequential constraints improve the consistency of road assignments [22,23], while trajectory preprocessing supports the extraction of informative motion patterns [24]. In road-network-assisted localization, visual odometry, wheel odometry, or short-term motion trajectories can instead be matched with OpenStreetMap data, polyline-based road networks, or topological maps, using probabilistic localization, particle filtering, pose graph optimization, or trajectory alignment [16,17,18,19,25,26,27]. Related studies have also represented motion through heading-length features and topology-constrained sequences, highlighting the value of structural context across successive observations [28,29,30]. Unlike conventional map matching, which typically uses approximate geographic observations, GNSS-denied initialization must infer the starting position from a locally referenced odometry trajectory and its absolute-heading information, without direct absolute position observations.

For initial localization without an absolute position reference, existing methods often reduce the search space by extracting local features from trajectories or road networks, such as turning points, intersections, line segments, or short trajectory fragments [25,26,27,28,29,30]. These features are easier to match than a complete trajectory, but they still face two limitations. First, standard polyline-based road maps mainly represent intersections, endpoints, and road connectivity, while internal direction-change and continuous-curvature structures along roads may not be adequately represented. Second, an individual turning point, a single line segment, or a short local fragment usually provides only partial geometric evidence and therefore has limited discriminative ability, especially in regular grids, dense intersections, or regions with repeated local patterns. Although sequence models and topological constraints can alleviate single-step matching ambiguity [31,32], an odometry trajectory without an absolute position reference still requires local structural units that jointly encode motion cues, together with a mechanism for evaluating the topology of successive correspondences.

To address these requirements, this paper proposes a topology-consistent trajectory-event matching method for vehicle initial localization under GNSS-denied conditions. Motivated by biological route navigation, the method represents self-motion as ordered local route structures and uses their sequential relations to resolve local ambiguity. Adjacent normal turning events and their intervening corridor form trajectory structure pairs; corresponding map-side structures are generated by augmenting internal direction-change points in the standard road-network input; successive matches are then connected under topological continuity before local spatial refinement.

The main contributions of this study are summarized as follows.

1. A trajectory structure-pair representation that integrates directional relationships, corridor-scale information, and internal motion morphology across adjacent normal turning events, providing a more discriminative structural description than isolated local features.
2. A map-side event-correspondence representation that augments internal direction-change points and curve-related structures in standard road-network maps, improving the correspondence between trajectory-side motion structures and map-side topological structures.
3. A topology-consistent initial localization mechanism that resolves local matching ambiguity through candidate-chain confirmation and subsequently recovers the continuous starting position through local spatial refinement.

The remainder of this paper is organized as follows. Section 2 presents the problem formulation and the overall framework. Section 3 describes the proposed trajectory-event topological localization method. Section 4 reports the experimental results and analysis. Section 5 discusses the applicability and limitations of the proposed method. Section 6 concludes the paper.

## 2. Problem Formulation and Overall Framework

This section formulates the problem of vehicle initial localization under GNSS-denied conditions and introduces the overall framework. The detailed construction of the enhanced map, trajectory structure pairs, candidate chains, and local trajectory-image refinement is presented in Section 3.

### 2.1. Problem Formulation

Let the standard road-network map covering the possible vehicle area be ℳ*_Ω_*, where *Ω* denotes a finite search region given by a coarse prior. This prior defines the regional map search range, while the vehicle road assignment and exact starting point are determined by the proposed localization process. The map data are converted into a local East-North-Up (ENU) coordinate frame for subsequent geometric computation with the trajectory.

The odometry trajectory during the initialization stage is denoted as

*T* = {*q_i_* = (*x_i_*, *y_i_*, *ψ_i_*, *s_i_*)∣*i* = 1, 2, ⋯, *N*} (1)

where (*x_i_*, *y_i_*) denotes the position of the *i*th trajectory point in the local odometry coordinate frame, *ψ_i_* denotes the vehicle heading, *s_i_* denotes the accumulated travel distance along the trajectory, and *N* is the number of trajectory points. The heading is an absolute heading referenced to geographic north, with clockwise rotation defined as positive. The trajectory coordinates are aligned according to this convention, and map-side road directions use the same convention. The shared heading reference aligns the trajectory and map orientations, so the initialization process estimates the planar starting position under GNSS denial.

The objective of this study is to estimate the initial position of the vehicle in the map coordinate frame, denoted by ${\hat{p}}_{0}$. Therefore, GNSS-denied initial localization can be formulated as

(2) $${\hat{p}}_{0}=F\left(M_{\Omega },T\right)$$

where ℱ(⋅) denotes the localization process consisting of map topology enhancement, trajectory-event construction, candidate retrieval, topological-consistency confirmation, and local spatial refinement. The localization process establishes correspondences between direction-change structures in the locally referenced odometry trajectory and topological structures in the road network.

The problem involves two main difficulties. The odometry trajectory provides only relative motion, so candidate roads must be inferred from structural correspondence with the map. Meanwhile, standard road-network maps may omit internal direction-change structures that are important for matching trajectory turns. The method therefore constructs discriminative trajectory structures and an enhanced map representation before correspondence search.

### 2.2. Overall Framework

**Figure 1:**
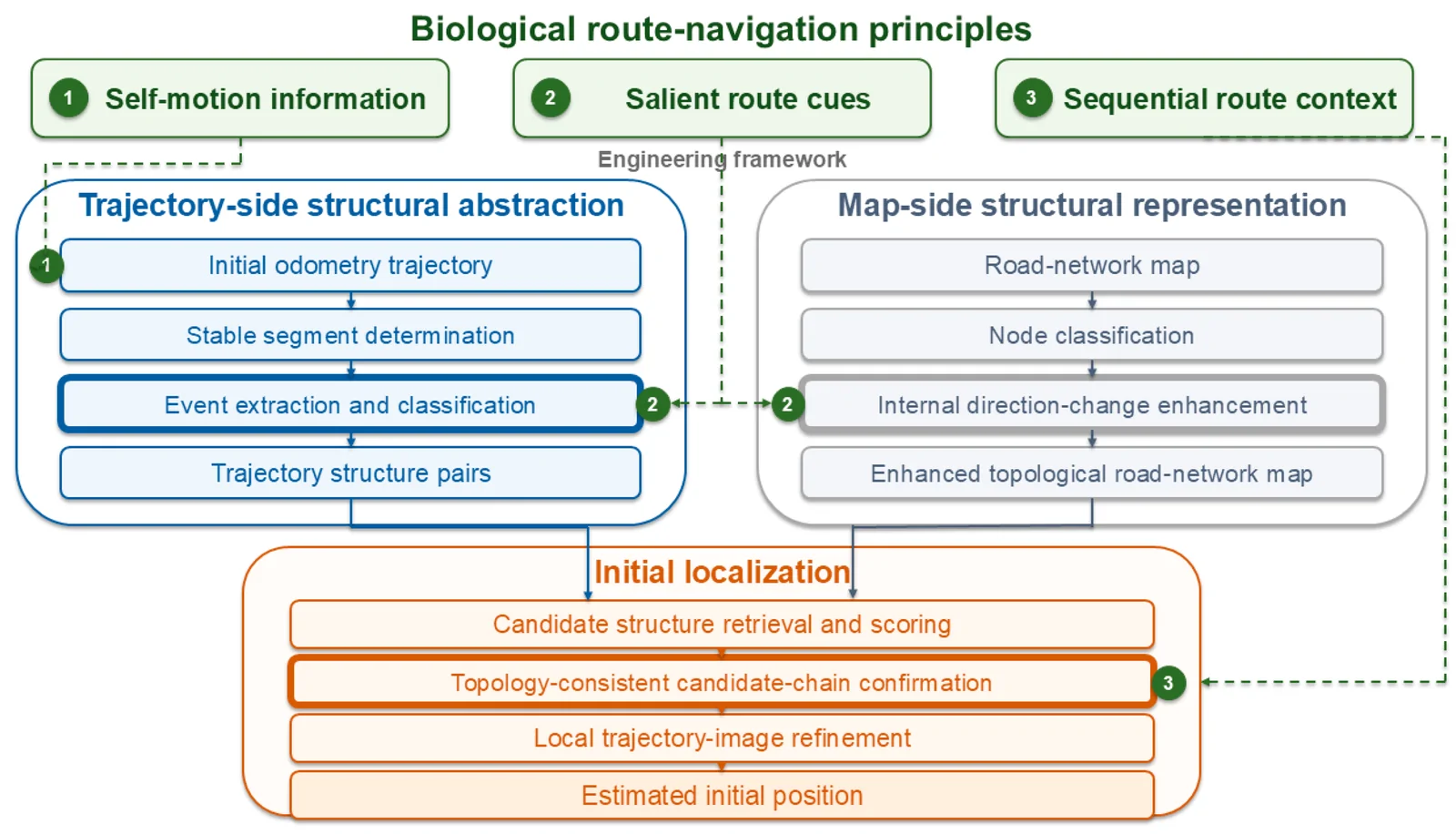
Overall framework of the proposed bio-inspired initialization method. Green boxes and dashed connectors indicate the biological navigation principles and their engineering correspondences; blue, gray, and orange groups indicate trajectory-side processing, map-side processing, and initial localization, respectively.

Figure 1 shows the overall workflow. Inspired by biological route navigation, the framework retains self-motion information from odometry, represents salient direction changes as local route structures, and preserves their sequential relations during candidate-chain confirmation. Given a standard road-network map and an initialization trajectory, the method constructs trajectory-side and map-side structures, retrieves local candidates, confirms a topology-consistent chain, and finally refines the starting position.

Local correspondence is evaluated from direction, scale, turning relation, and corridor morphology. Topological continuity then accumulates these local correspondences along the travelled route, providing the discrete map association used for continuous position refinement [8,9,10,11,12,13,14,15].

## 3. Proposed Method

**Figure 2:**
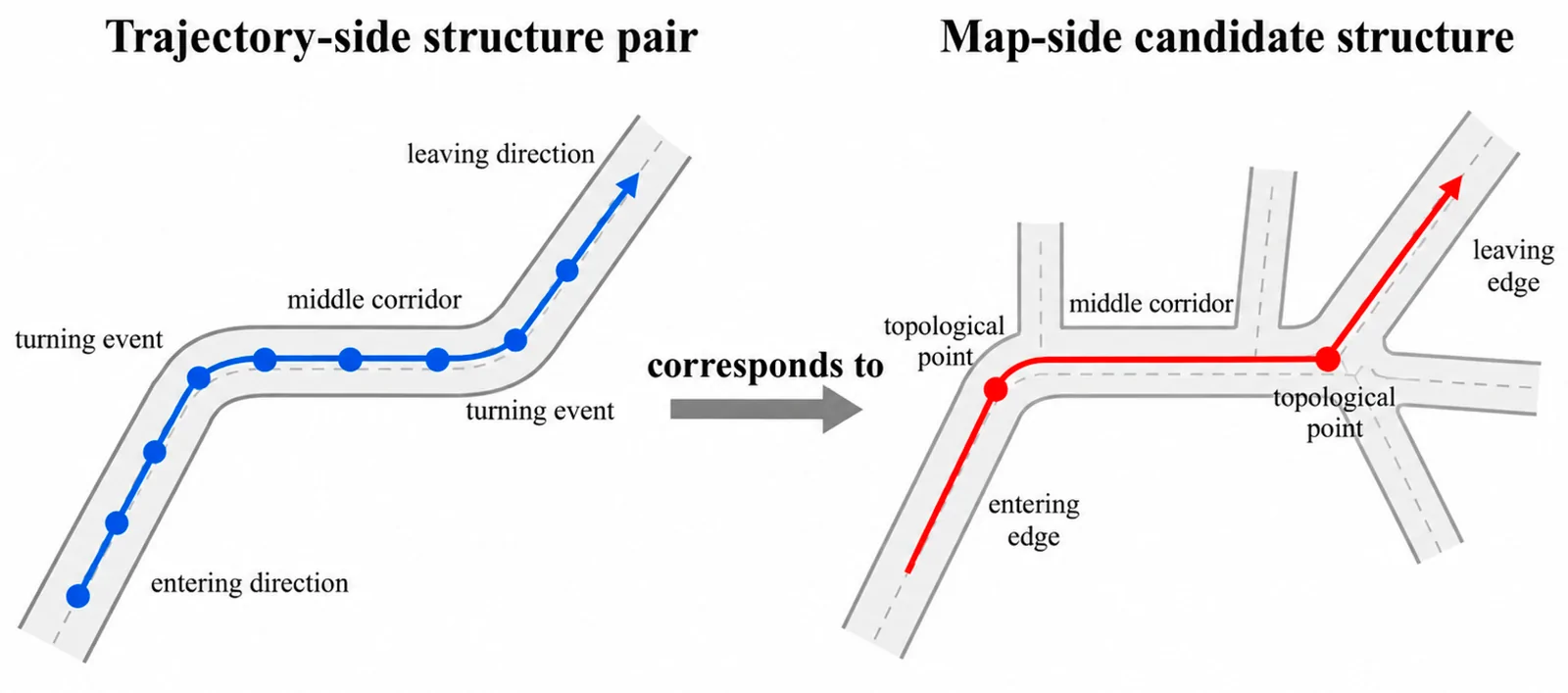
Basic correspondence between trajectory-side and map-side structures. Blue marks the trajectory-side structure, red marks the corresponding map-side structure, and gray indicates the road geometry.

This section describes the proposed trajectory-event topological localization method. Figure 2 illustrates the basic correspondence between the trajectory-side structure pair and the map-side candidate structure. The trajectory side is represented by adjacent normal turning events, whereas the map side is represented by an entering edge, a middle corridor, and a leaving edge in the enhanced road network. The detailed construction of these structures is introduced in the following subsections.

### 3.1. Map Topology Enhancement

A raw polyline-based road network usually explicitly provides only intersections and endpoints, whereas internal direction-change structures along roads may not be preserved. To address this limitation, a map-side topology enhancement mechanism is introduced to construct an enhanced directed topology representation for direction-sensitive matching.

First, the regional road-network map is converted into a local coordinate frame, and nodes are classified from connectivity and local geometric deflection into intersection nodes, endpoint nodes, internal bend nodes, and ordinary shape nodes. For a degree-2 node, its local deflection angle is defined from the two adjacent road segments. A node exceeding the bend threshold is labeled as a bend node and is promoted to an internal turning point when it also exceeds the stronger turning threshold. This distinction retains meaningful direction-change anchors while avoiding an excessive number of topological nodes caused by minor polyline fluctuations.

The promoted internal turning points, together with intersection nodes, endpoint nodes, and necessary virtual nodes, form the enhanced topological point set.

Based on the enhanced topological point set *TP*^+^, the original road network is topologically contracted to construct a directed topological edge set *EG*. A single directed topological edge is defined as

*EG* = (*TP_s_*, *SP*_1:*N*_, *RS*_1:*N*+1_, *TP_e_*) (3)

where *TP_s_* and *TP_e_* denote the starting and ending topological points, respectively; {*SP*_1:N_} denotes the sequence of internal shape points; and {*RS*_1:N+1_} denotes the ordered sequence of road segments. To support the following direction-sensitive matching process, both directions of an edge are retained as valid directed edges. After topology contraction, the geometric information and connectivity of the map are organized into directed structural units that can be directly used for candidate construction.

**Figure 3:**
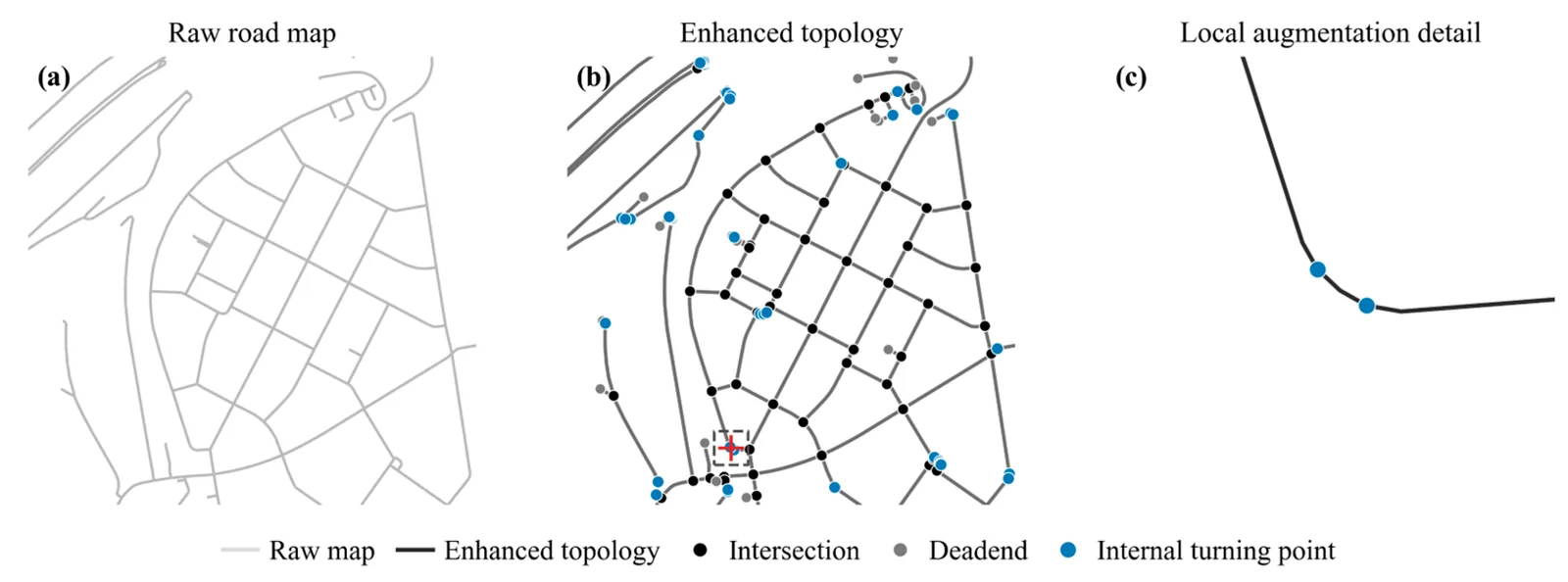
Map topology enhancement: (**a**) raw road-network map; (**b**) enhanced topological representation; (**c**) local augmentation of internal direction-change points. Gray lines indicate the raw map, black lines the enhanced topology, black dots intersections, gray dots endpoints, and blue dots internal turning points.

Figure 3 shows the map topology enhancement process. Internal road nodes satisfying the threshold condition are promoted to internal turning points, and the resulting enhanced topology provides map-side anchors for structural matching.

To improve geometric stability, road segments longer than the threshold are linearly interpolated. Continuous-curvature edges are then identified according to the cumulative turning angle, edge length, and average curvature within directed topological edges. For a directed edge, let *Θ_j_* and *L_j_* denote its cumulative turning angle and length, respectively. The average curvature is defined as

(4) $$\kappa_{j}=\frac{\Theta_{j}}{L_{j}}$$

When the cumulative turning angle, average curvature, and edge length meet their respective criteria in Table 2, the edge is identified as a continuous-curvature edge for subsequent middle-corridor morphology evaluation.

*M*^+^ = {*TP*^+^, *EG*} (5)

After the above processing, the enhanced map representation is denoted as *M*^+^ and is used as the basis for constructing and evaluating map-side candidate topological structure pairs.

### 3.2. Trajectory-Event Extraction and Structure-Pair Construction

Local fluctuations and accumulated drift make direct association between a raw odometry trajectory and map roads unstable. In insect route navigation, familiar-route following is supported by locally recognizable route information and its sequential organization [8,9,10,11,12,13,14]. Following this idea, the odometry trajectory is organized into structure pairs formed by adjacent normal turning events and their intervening corridor, which serve as the basic matching units.

First, the inertial odometry trajectory is preprocessed to determine a stable initial trajectory segment. A direction-change response is then computed in the distance domain, and direction-change active intervals are extracted. The *k*th active interval is denoted as

(6) $$A_{k}=\left[s_{k}^{b},s_{k}^{e}\right]$$

where $s_{k}^{b}$ and $s_{k}^{e}$ denote the starting and ending positions of the active interval in the distance domain, respectively. Spatially adjacent active intervals belonging to the same direction-change process are further merged to obtain more stable direction-change candidate sections. The entering and exiting of an active interval are controlled by the thresholds *τ_in_* and *τ_out_*, which suppress false event triggers caused by local jitter while preserving the completeness of true direction-change processes.

Based on the active intervals, the trajectory is divided into straight segments, continuous-curvature segments, normal turning events, and U-turn events according to interval length, direction-change magnitude, and local morphology. The *i*th turning event is denoted as

*E_i_* = (*p_i_*, *θ_i_*, *c_i_*) (7)

where *p_i_* denotes the representative point of the event, *θ_i_* denotes the direction-change angle, and *c_i_* denotes the event type. Normal turning events are used as anchors for constructing trajectory structure pairs, whereas U-turn events are excluded from pair construction to avoid unstable matching.

**Figure 4:**
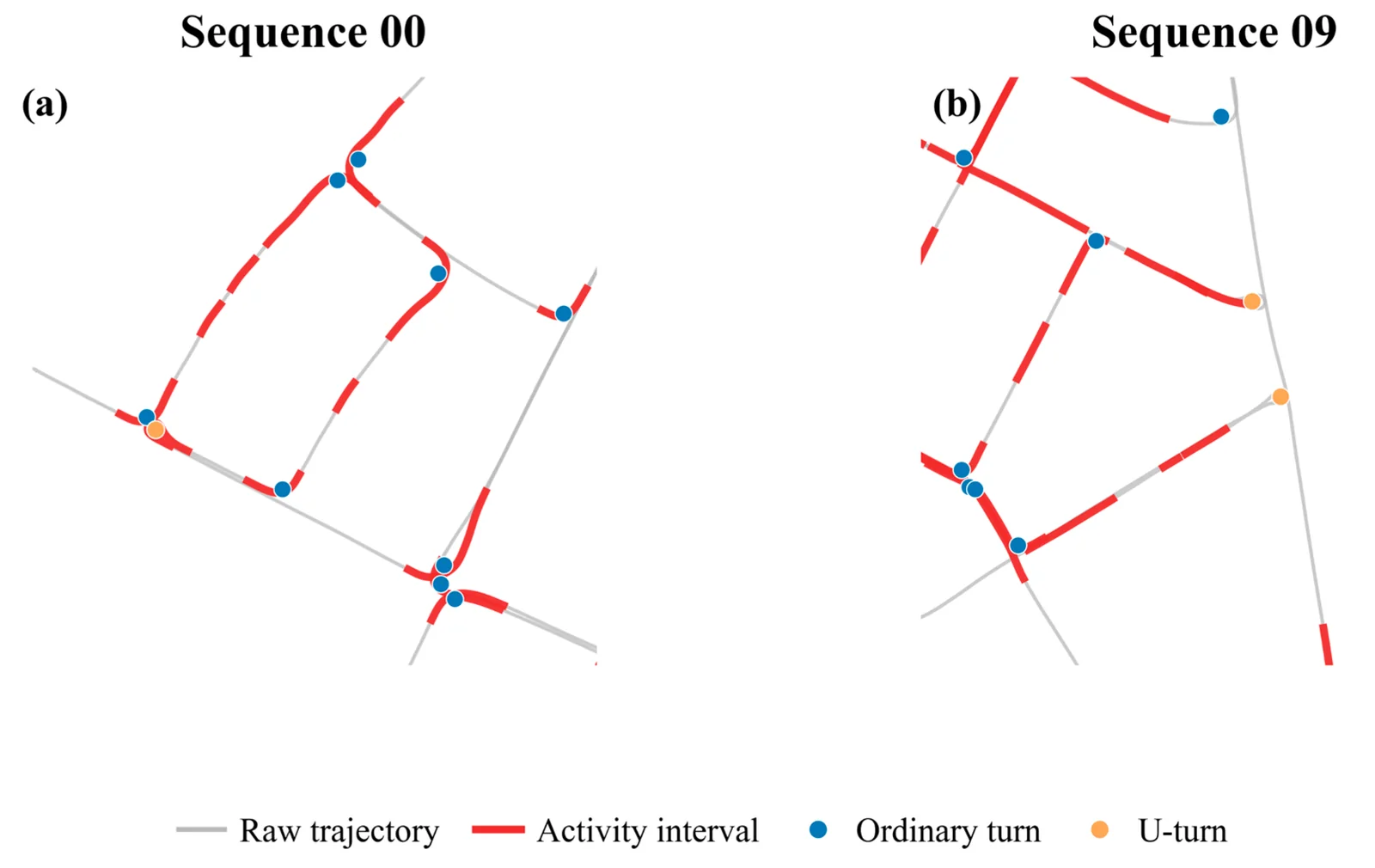
Trajectory-event extraction results for (**a**) Seq. 00 and (**b**) Seq. 09. Gray lines show the raw trajectory, red segments the direction-change active intervals, blue dots normal turns, and orange dots U-turns.

Figure 4 shows the trajectory-event extraction result. Direction-change active intervals indicate segments with significant heading variation, and normal turning events serve as anchors for structure-pair construction.

On this basis, turning events are scanned in chronological order. A trajectory structure pair is constructed only when two adjacent events are both normal turning events. If a U-turn or another abnormal direction-change behavior exists between them, the corresponding structure pair is not constructed. The *i*th trajectory structure pair is defined as

*Z_i_* = (*ψ_p_*_, *i*_, *ψ_m_*_, *i*_, *ψ_o_*_, *i*_, *L*_mid,*i*_, *F*_mid,*i*_) (8)

where *ψ_p_*_,*i*_, *ψ_m_*_,*i*_, *ψ_o_*_,*i*_, and *L*_mid,*i*_ denote the entering direction, middle-corridor direction, leaving direction, and middle-corridor length, respectively. The internal morphology of the middle corridor is further described by

*F*_mid,*i*_ = (*ψ_h_*_,*i*_, *ψ_t_*_,*i*_, *A_i_*, *S_i_*, *κ_i_*,*σ_ψ_*_,*i*_, *R_ψ_*_,*i*_) (9)

where *ψ_h_*_,*i*_ and *ψ_t_*_,*i*_ denote the local directions in the front and rear parts of the middle corridor, respectively; *A_i_* and *S_i_* denote the accumulated absolute turning angle and accumulated signed turning angle, respectively; *κ_i_* denotes the average curvature; *σ_ψ_*_,*i*_ denotes the heading fluctuation; and *R_ψ_*_,*i*_ denotes the heading range. To avoid unstable matching caused by extremely short corridors, only adjacent normal turning-event pairs with a middle-corridor length not smaller than the threshold *L*_min_ are retained.

After this processing, the raw odometry trajectory is represented as an ordered sequence of trajectory structure pairs, which serves as the basic unit for subsequent map-side candidate construction and matching.

### 3.3. Topology-Consistent Initial Localization

After map-side and trajectory-side structures are obtained, initialization is performed by retrieving local map-side candidates, confirming a topologically consistent candidate chain, and refining the continuous starting position.

#### 3.3.1. Map-Side Candidate Construction and Matching Score

To correspond to the trajectory structure pair *Z_i_*, map-side candidate topological structure pairs are constructed based on the enhanced map representation *M*^+^. The *j*th candidate topological structure pair is denoted as

(10) $$MZ_{j}=\left(e_{j}^{\mathrm{in}},TP_{u,j},C_{j},TP_{v,j},e_{j}^{\mathrm{out}}\right)$$

where $e_{j}^{\mathrm{in}}$ and $e_{j}^{\mathrm{out}}$ denote the entering and leaving edges, respectively; *TP_u_*_,*j*_ and *TP_v_*_,*j*_ denote the starting and ending topological points, respectively; and *C_j_* denotes the middle corridor connecting them. This representation is consistent with the trajectory structure pair in form, as both contain an entering direction, a middle corridor, and a leaving direction, and therefore supports local matching evaluation in a unified framework.

For each map-side candidate topological structure pair, its matching attributes are further extracted and denoted as

(11) $$G_{j}=\left(\psi_{p,j}^{m},\psi_{m,j}^{m},\psi_{o,j}^{m},L_{\mathrm{mid},j}^{m},F_{\mathrm{mid},j}^{m}\right)$$

where $\psi_{p,j}^{m}$, $\psi_{m,j}^{m}$, and $\psi_{o,j}^{m}$ denote the entering direction, middle-corridor direction, and leaving direction, respectively; $L_{\mathrm{mid},j}^{m}$ denotes the middle-corridor length; and $F_{\mathrm{mid},j}^{m}$ denotes the internal morphology feature of the middle corridor. These attributes jointly describe the directional relationships, scale information, and internal geometry of the candidate structure.

The consistency between a trajectory structure pair and a map-side candidate structure pair is then evaluated from four aspects: direction, length, local turning relationship, and internal morphology of the middle corridor. The local turning relationships on the trajectory side and the map side are first defined as

*Δψ*_1,*i*_ = *ψ_m_*_,*i*_ − *ψ_p_*_,*i*_, Δ*ψ*_2,*i*_ = *ψ_o_*_,*i*_ − *ψ_m_*_,*i*_ (12)

(13) $$\Delta \psi_{1,j}^{m}=\psi_{m,j}^{m}-\psi_{p,j}^{m},\;\Delta \psi_{2,j}^{m}=\psi_{o,j}^{m}-\psi_{m,j}^{m}$$

Accordingly, the three-segment direction differences, length consistency, local turning-relationship differences, and accumulated absolute turning-angle difference of the middle corridor are defined.

The trajectory headings and map-side road directions share the same north-referenced, clockwise-positive convention, allowing their directional differences to be computed directly after angular normalization during candidate retrieval.

(14) $$\begin{array}{c} e_{p}=\mathrm{wrap}\left(\psi_{p,i}-\psi_{p,j}^{m}\right),\\ e_{m}=\mathrm{wrap}\left(\psi_{m,i}-\psi_{m,j}^{m}\right),\\ e_{o}=\mathrm{wrap}\left(\psi_{o,i}-\psi_{o,j}^{m}\right) \end{array}$$

(15) $$r_{L}=\frac{\min\left(L_{\mathrm{mid},i},L_{\mathrm{mid},j}^{m}\right)}{\max\left(L_{\mathrm{mid},i},L_{\mathrm{mid},j}^{m}\right)}$$

(16) $$\begin{array}{c} e_{\Delta 1}=\mathrm{wrap}\left(\Delta \psi_{1,i}-\Delta \psi_{1,j}^{m}\right),\\ e_{\Delta 2}=\mathrm{wrap}\left(\Delta \psi_{2,i}-\Delta \psi_{2,j}^{m}\right) \end{array}$$

(17) $$e_{A}=A_{i}-A_{j}^{m}$$

where wrap denotes the angle normalization operator. Candidate evaluation is performed in two stages. Feasibility constraints first remove candidates with clearly inconsistent direction, length, or turning relations. The remaining candidates are scored using direction, length, turning relation, and corridor morphology, and the highest-ranked candidates are retained for chain construction. This preserves plausible local hypotheses while limiting the subsequent search scale. The parameter settings are given in Section 4.1.

#### 3.3.2. Candidate-Chain Confirmation

Candidate-chain confirmation is introduced because a single local structure may match several geometrically similar road segments. Successive structures provide stronger evidence: their map-side correspondences must preserve the ordering and connectivity of the travelled route. Local candidates are therefore connected into chain hypotheses that satisfy topological continuity. A candidate chain is denoted as

*C* = (*MZ*_1_, *MZ*_2_, ⋯, *MZ_K_*) (18)

where *MZ_k_* denotes the map-side candidate topological structure pair corresponding to the *k*th trajectory structure pair. Here, *K* is the number of map-side candidate structure pairs in the chain. If two adjacent candidates satisfy the topological-consistency constraint, they are allowed to be connected into the same candidate chain. For strict connection, the basic condition for two adjacent candidates is expressed as

*TP_v_*_,*k*_ = *TP_u_*_,*k*+1_ (19)

that is, the ending topological point of the *k*th candidate is identical to the starting topological point of the (*k* + 1)th candidate. A limited bridge connection is allowed for a single missing or unmatched structure pair when the preceding and succeeding candidates remain topologically consistent. This tolerance handles occasional event omissions without weakening the overall route-level constraint.

**Figure 5:**
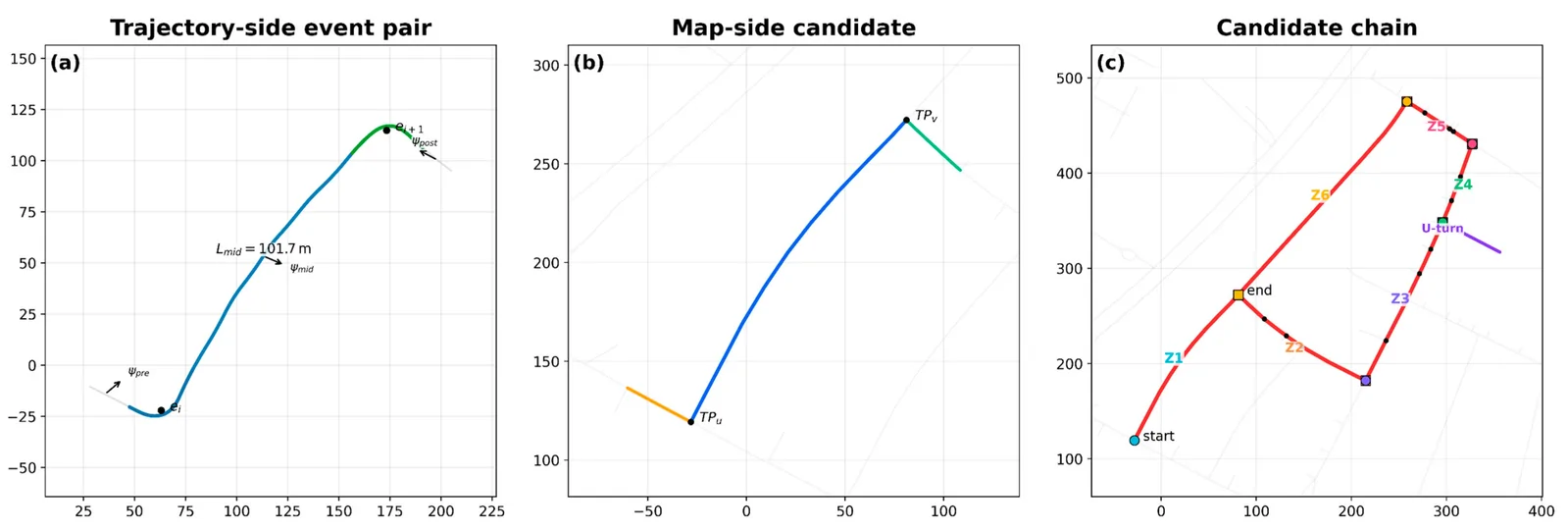
Topology-consistent candidate-chain construction: (**a**) trajectory-side structure pair; (**b**) map-side candidate structure; (**c**) candidate chain formed from successive correspondences. In (**b**), orange, blue, and green indicate the entering edge, middle corridor, and leaving edge, respectively. In (**c**), the red path shows the candidate chain, colored labels and markers distinguish successive structure pairs, and the purple annotation identifies a U-turn.

As illustrated in Figure 5, local candidate matches are promoted to a chain-level explanation by enforcing shared topological points or bridge constraints among adjacent structures.

A beam search strategy is used to control the growth of candidate chains. At each step, only the highest-scoring partial chains are retained, so the beam width balances hypothesis coverage against computational cost. The retained local-candidate count and beam width are summarized in Section 4.1.

After candidate chains are generated, chain-level evaluation is further performed. The comprehensive score of a candidate chain *C* is denoted as

(20) $$J_{C}=\sum_{k=1}^{K}S_{Z}\left(Z_{k},MZ_{k}\right)-\lambda_{m}N_{m}-\lambda_{b}N_{b}$$

where *S_Z_*(*Z_k_*, *MZ_k_*) denotes the local matching score between the *k*th trajectory structure pair and the corresponding candidate; *N_m_* denotes the number of missing matches in the chain; *N_b_* denotes the number of bridge connections; and *λ_m_* and *λ_b_* are the corresponding penalty coefficients. The score therefore favors chains that combine strong local matches with sustained topological continuity.

Finally, the candidate chain with the highest comprehensive score is selected as the initialization result in the discrete topological sense. To evaluate the confidence of the result, the score gap between the best and second-best candidate chains is calculated. A larger score gap indicates that the current result has stronger discrimination against competing candidates, whereas a smaller score gap indicates that strong ambiguity remains in the local road network.

#### 3.3.3. Local Trajectory-Image Refinement

After candidate-chain confirmation, the discrete topological correspondence of the initial trajectory in the map can be determined. To further estimate the continuous starting position, local trajectory-image refinement (TI refinement) is performed near the first confirmed structure.

Let the first confirmed candidate topological structure pair be

*MZ*_1_ = (*EG*^in^, *TP_u_*, *C_u_*_→*v*_, *TP_v_*, *EG*^out^) (21)

Based on the first confirmed candidate structure, a local map image and a corresponding trajectory image are generated in the same local coordinate frame and grid resolution. The local road structure is discretized into the map image, and the trajectory segment associated with the first structure pair is rasterized into the trajectory image. The discrete structural correspondence is then transformed into a local image-alignment problem.

The trajectory image is translated within a local search range, and the displacement with the best map-image overlap is taken as the continuous position correction. Because the odometry trajectory and map directions share the same absolute-heading reference, the refinement searches only translation. The search range, grid resolution, and step size jointly determine refinement accuracy and computational cost; their settings are given in Section 4.1.

(22) $${\hat{p}}_{0}=p_{r}+\Delta p$$

where *p_r_* denotes the reference position determined by the first confirmed map structure, and *Δp* denotes the translation correction obtained by local image alignment. Thus, candidate-chain confirmation provides the discrete topological result, and TI refinement further estimates the starting position in continuous space.

**Figure 6:**
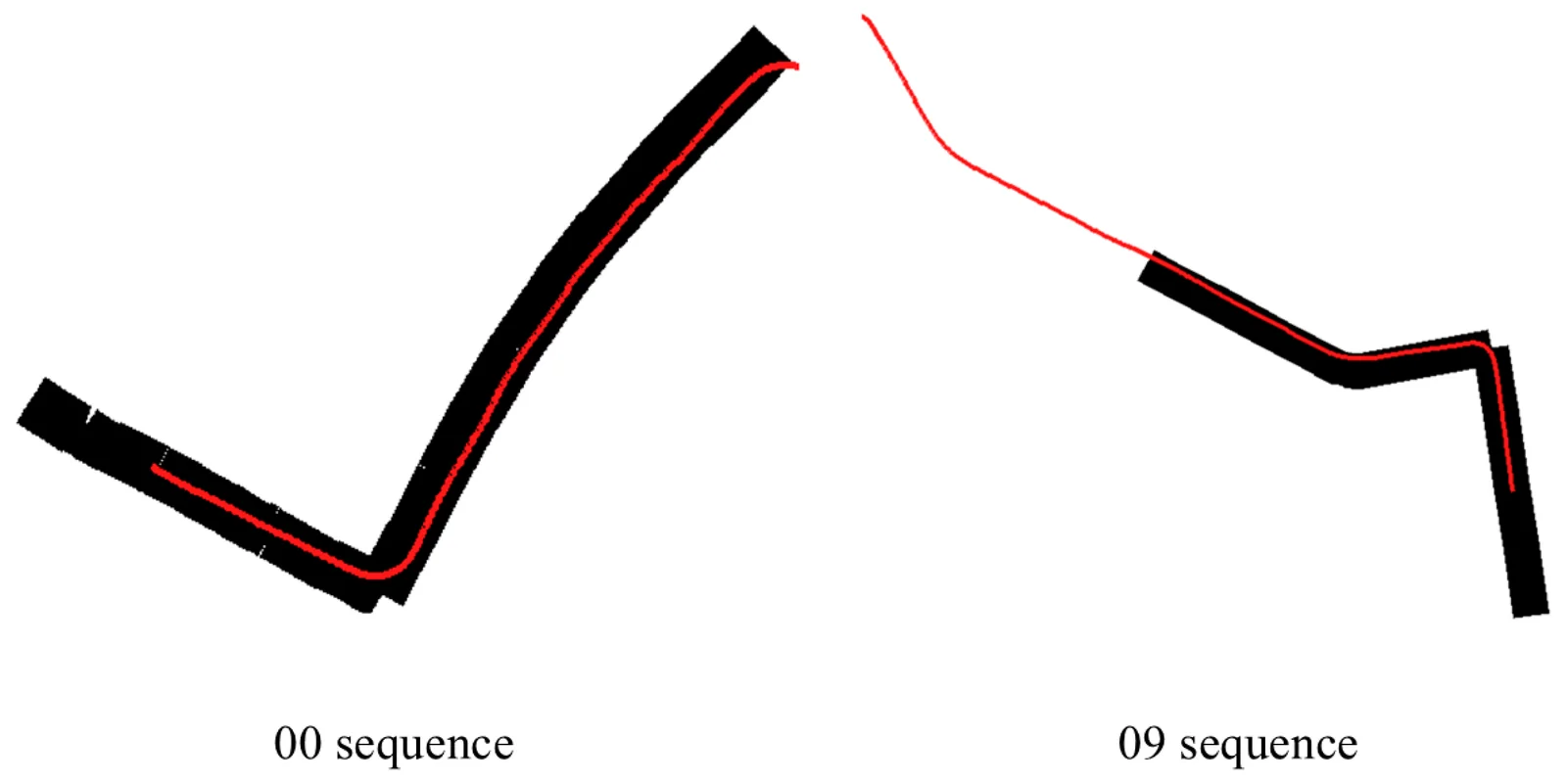
Local trajectory-image refinement for representative cases from Seq. 00 (**left**) and Seq. 09 (**right**). Black regions represent the rasterized map roads, and red curves represent the translated trajectory segments.

Figure 6 shows an example of local trajectory-image refinement, where the local trajectory segment is translated within a limited search range to obtain the continuous starting-position correction. Table 1 summarizes the complete initialization sequence from structural representation to continuous position refinement.

## 4. Experiments

### 4.1. Experimental Setup

To evaluate the initial localization performance of the proposed method, experiments are conducted on public road-scene data. The external map input is a standard OpenStreetMap road-network polyline map, from which the algorithm automatically generates the enhanced topological representation used for matching. Regional maps are converted into a local ENU coordinate frame. Vehicle trajectories are provided by odometry data from KITTI-360 sequences. Sequences 00, 08, and 09 are selected for qualitative and quantitative evaluation, covering regular grid-like road networks, roads with more internal direction changes, and regions with strong local structural similarity.

The proposed method is evaluated in terms of initialization accuracy, adaptability to different starting points, candidate retrieval effectiveness, and computational cost. Initialization accuracy is the two-dimensional Euclidean distance between the estimated and ground-truth starting positions in the ENU plane, with ground truth obtained from the KITTI-360 reference GNSS/INS poses. A valid estimate with an error of 10 m or less is counted as successful. This threshold is used as a gross-failure criterion because larger errors generally indicate an incorrect road or candidate-chain association rather than the residual of local refinement. SR@5 m denotes the fraction of all initialization trials that yield a valid estimate with an error of 5 m or less. The mean, median, standard deviation, and range of successful-trial errors are also reported; failed and timed-out trials are excluded from the error statistics, with 10 min used as the timeout limit.

Unless otherwise specified, all sequences use the same parameter setting, as summarized in Table 2. The matching process follows a two-stage design: local filters retain multiple plausible candidates, and topological chain confirmation subsequently imposes the stronger sequential constraint. After a discrete chain is confirmed, local TI refinement is performed with translation-only search.

For the multi-start robustness experiment, 45 starting positions are sampled at approximately uniform spatial intervals along the usable portion of each sequence, yielding intervals of approximately 152.2 m, 112.9 m, and 178.6 m for sequences 00, 08, and 09, respectively. The sampling rule is independent of local turning-event density, and only the odometry trajectory after each selected start is supplied to the initialization pipeline, giving 135 trials in total. For candidate-retrieval analysis, the first eight valid trajectory structure pairs of each sequence are used. Successful initialization typically requires about four to six structure pairs for chain confirmation, so these eight pairs cover the principal retrieval interval while later pairs are progressively less likely to participate. The ground-truth map-side candidate is obtained from the reference GNSS/INS trajectory and manually checked for consistency in position, road direction, and topology.

All experiments were conducted on a laptop equipped with an AMD Ryzen 5 4600U processor (Advanced Micro Devices, Inc., Santa Clara, CA, USA) and 16 GB of RAM. The program was implemented in Python 3.13. Runtime statistics include only the online initialization stage, including trajectory-event extraction, candidate retrieval, candidate-chain confirmation, and local TI refinement, and exclude offline map construction. The average number of event pairs required for confirmation and the average relative confirmation margin are also reported to evaluate chain-level confirmation efficiency and discrimination.

### 4.2. Overall Initialization Results

**Figure 7:**
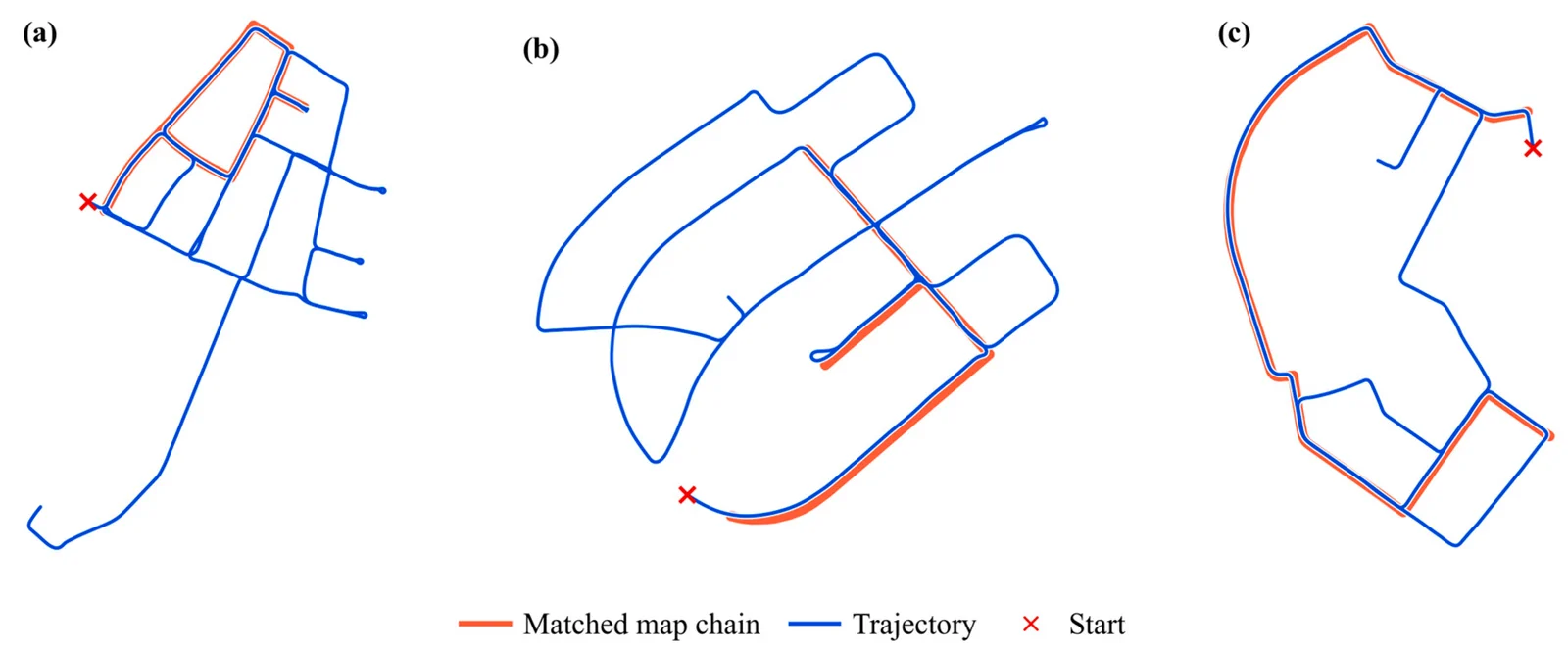
Representative initialization results on KITTI-360: (**a**) Seq. 00; (**b**) Seq. 08; (**c**) Seq. 09. The blue curve is the odometry trajectory, the red polyline the confirmed map-side candidate chain, and the red cross the estimated starting position after TI refinement.

Figure 7 illustrates representative initialization results for Seq. 00, Seq. 08, and Seq. 09 in panels (a–c), respectively. The blue curve represents the odometry trajectory used for initialization, the red polyline the confirmed map-side candidate chain, and the red cross the starting position after TI refinement. The results illustrate how chain confirmation and local refinement recover a continuous map-referenced position across different road layouts.

Table 3 summarizes representative road-network-assisted localization results reported in prior work together with the results of the proposed method. The compared methods use different sensor inputs, map representations, trajectory processing strategies, and implementation settings, so Table 3 provides a contextual reference for their reported accuracy levels. The values for the proposed method correspond to the representative starts shown in Figure 7 and yield a mean error of 1.95 m. Variability with respect to the starting position is evaluated separately in Section 4.3.

### 4.3. Robustness Under Different Starting Points

**Figure 8:**
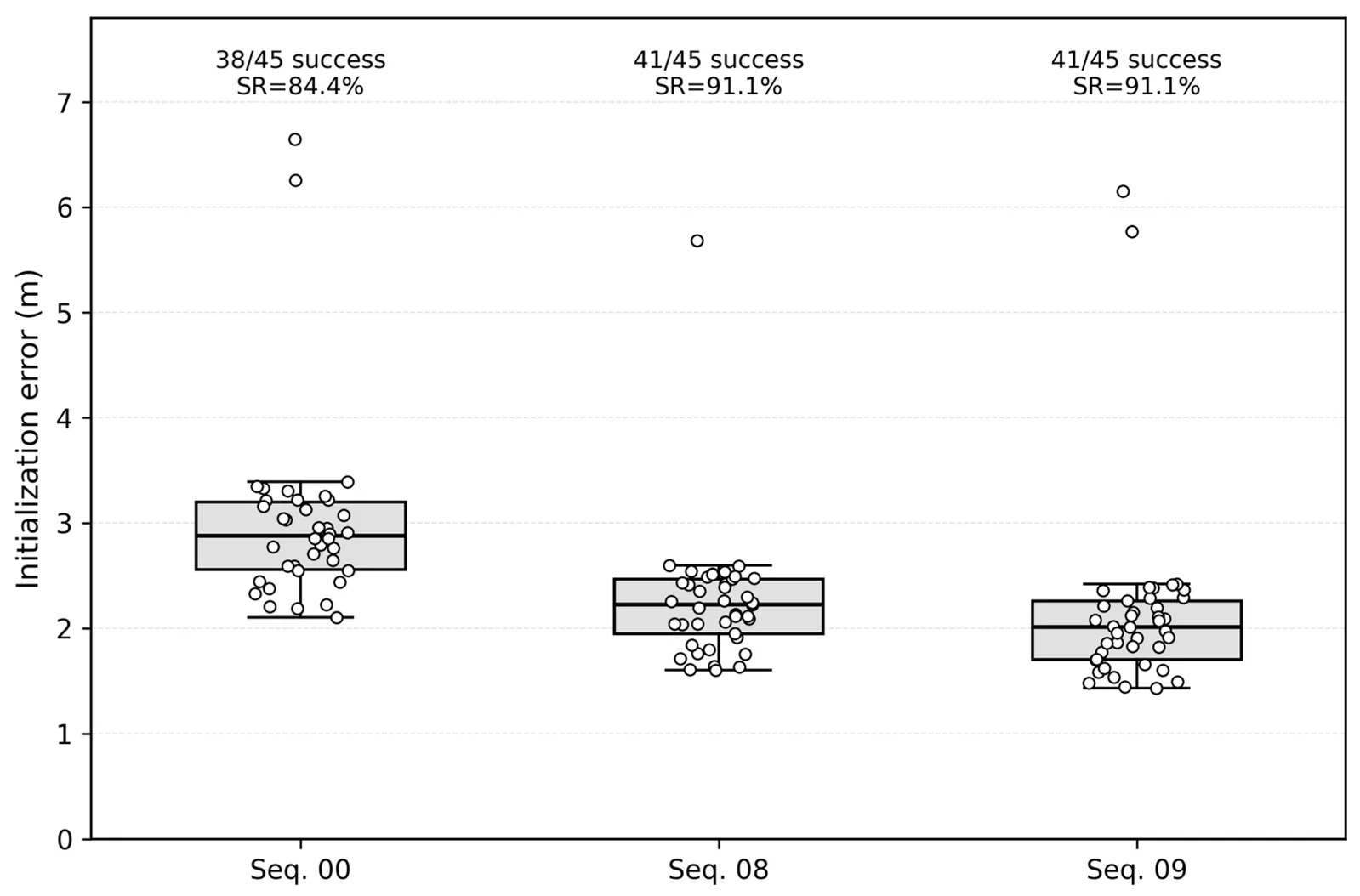
Distribution of localization errors across successful multi-start trials. Dots show individual successful trials; annotations above each sequence give the success counts and success rates under the 10 m criterion. Detailed statistics, including SR@5 m, are reported in Table 4.

To evaluate sensitivity to the starting position, 45 systematically sampled starts are used on each of the three sequences, giving 135 trials in total. Table 4 reports the success rate, SR@5 m, and error statistics over successful trials, while Figure 8 visualizes the corresponding successful-trial error distributions. The method succeeds in 38/45, 41/45, and 41/45 trials in sequences 00, 08, and 09, respectively, corresponding to an overall success rate of 88.9%. The overall SR@5 m is 85.2%, showing that most successful initializations remain within a relatively small error range.

Among successful trials, the mean errors in sequences 00, 08, and 09 are 3.01 m, 2.25 m, and 2.15 m, with medians of 2.88 m, 2.22 m, and 2.01 m, respectively. The corresponding standard deviations are 0.90 m, 0.63 m, and 0.92 m. The average numbers of structure pairs required for confirmation are 5.47, 4.20, and 4.63, while the average confirmation margins are 14.48, 30.37, and 28.77. Sequence 00 therefore requires more structural evidence and exhibits a smaller separation from competing chains than sequences 08 and 09. Figure 8 shows the same tendency: the successful-trial errors remain concentrated near the low-meter range for all three sequences, while Seq. 00 has a higher central error level and a broader upper tail.

The required observation length varies across the three sequences. Successful trials require average trajectory lengths of approximately 1.91 km, 0.99 km, and 0.86 km in sequences 00, 08, and 09, respectively, while failures are concentrated in long-straight or sparse-event regions and cases with weak separation between competing candidate chains. The average confirmation margin of failed trials is about 6–7, markedly below that of successful trials. These results indicate robustness to starting-position variation within the evaluated road scenarios and identify sparse route structure as an important condition affecting reliable initialization.

### 4.4. Candidate Retrieval Effectiveness and Computational Cost

**Figure 9:**
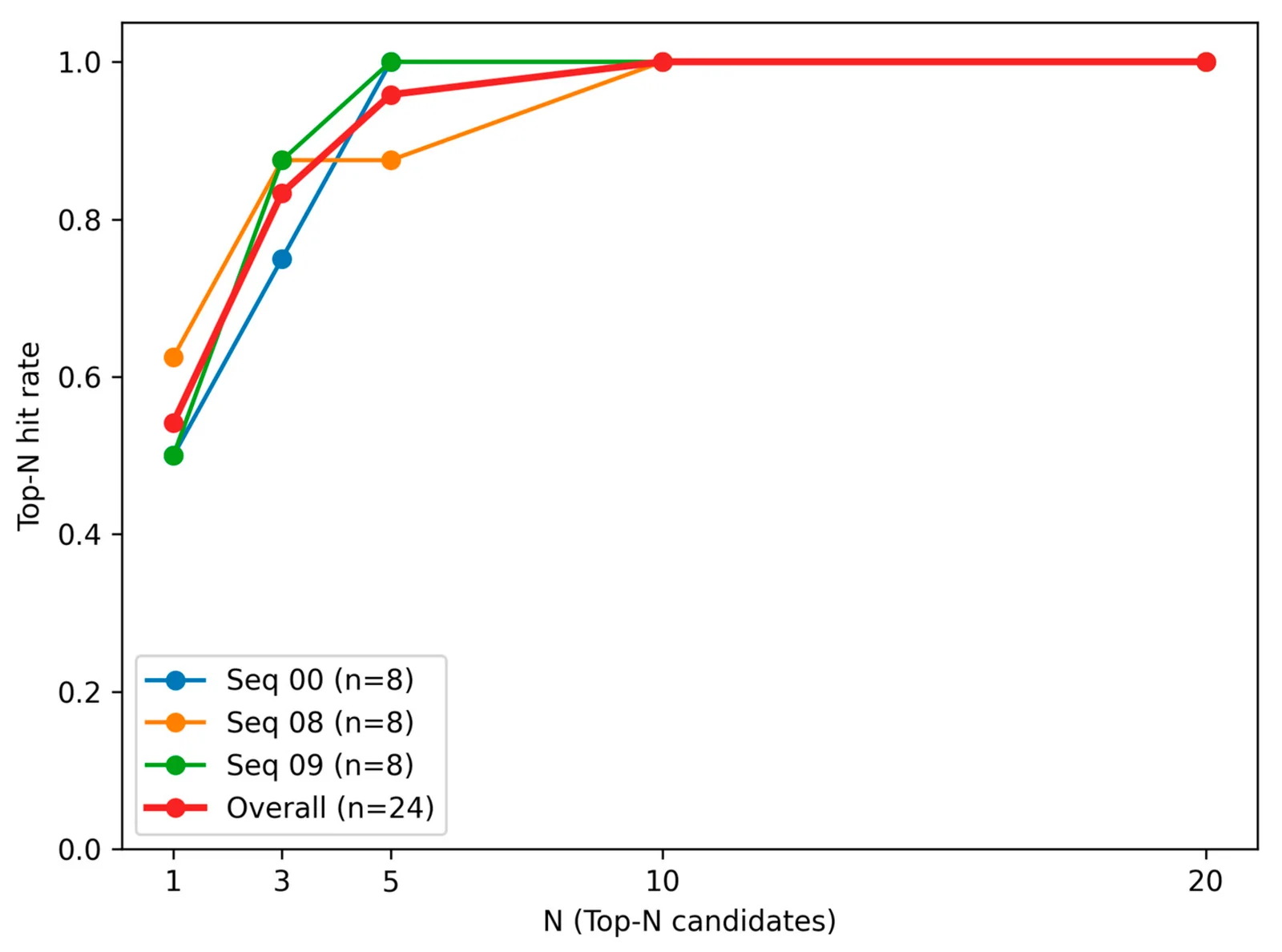
Top-*N* hit rate of the ground-truth map-side candidate over the first eight valid trajectory structure pairs of each sequence. Blue, orange, and green indicate Seq. 00, Seq. 08, and Seq. 09, respectively; red indicates the overall result.

Figure 9 reports the Top-*N* hit rate of the ground-truth map-side candidate over the first eight valid trajectory structure pairs of each sequence. A hit is recorded when the ground-truth candidate appears among the top *N* candidates. Seq. 00 and Seq. 09 reach a hit rate of 1.0 at *N* = 5, Seq. 08 reaches 1.0 at *N* = 10, and the overall rate reaches 1.0 at *N* = 10. The lower Top-1 rates show that several locally plausible candidates often coexist, which motivates the retention of multiple hypotheses for chain-level confirmation.

**Figure 10:**
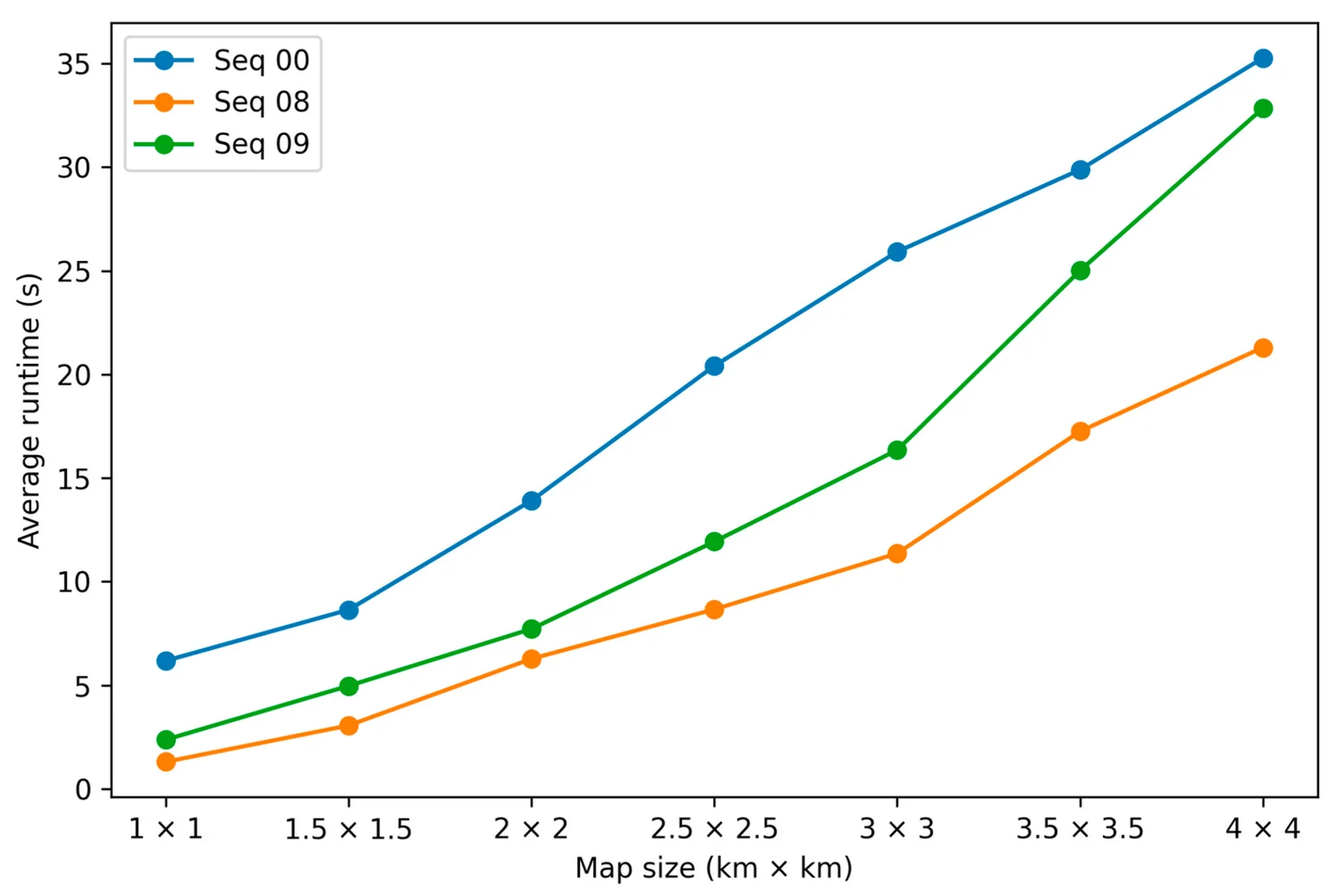
Average online initialization runtime versus map size for the three test sequences. Blue, orange, and green indicate Seq. 00, Seq. 08, and Seq. 09, respectively.

Figure 10 shows the runtime trend over the full set of tested map sizes, while Table 5 gives a compact stage-wise breakdown at four representative sizes (1 × 1, 2 × 2, 3 × 3, and 4 × 4 km). The average online initialization time increases from 3.28 s at 1 × 1 km to 29.80 s at 4 × 4 km. Across the same representative sizes, the mean number of map candidate structures increases from 451 to 7218 and candidate-chain expansions from approximately 730 to 1736. Candidate retrieval time rises from 0.77 s to 8.73 s, while candidate-chain confirmation rises from 1.80 s to 20.36 s and remains the dominant online cost. TI refinement changes little because it is confined to the local neighborhood of the confirmed chain.

### 4.5. Ablation Study

To analyze the contribution of each key module, ablation experiments remove or replace map-side internal direction-change augmentation, trajectory structure-pair construction, candidate-chain confirmation, and local TI refinement while keeping the remaining settings unchanged. Table 6 reports localization errors for successful cases, while ‘Fail’ denotes an invalid initialization or an error exceeding the 10 m threshold and ‘Timeout’ denotes a trial exceeding 10 min. Failures and timeouts are presented as categorical outcomes, and the numerical error statistics are calculated from successful cases.

After removing map-side internal direction-change augmentation, the method succeeds only in Seq. 00 and fails in Seq. 08 and Seq. 09. This indicates that internal direction-change points provide essential map-side anchors in scenes containing road bends or continuous direction changes and improve the completeness of candidate structures corresponding to trajectory structure pairs. The single-turning-event variant causes timeouts in Seq. 00 and Seq. 09, mainly because a single turning event contains only local direction-change information and cannot jointly constrain the entering direction, middle corridor, and leaving direction. As a result, many candidates may be generated in regions with similar local structures or regular grid-like road patterns.

The no-chain-confirmation variant fails in all three sequences. The result shows that topological continuity among adjacent structures is important for suppressing mismatches produced by locally high-scoring candidates. The no-TI-refinement variant preserves the topological correspondence but increases the average error to 7.77 m. Candidate-chain confirmation therefore determines the discrete topological position, while local trajectory-image refinement improves the continuous starting-position estimate.

Overall, the ablation results show distinct roles for the four main modules: map-side augmentation improves candidate completeness, structure pairs improve local discrimination, chain confirmation stabilizes the discrete topological correspondence, and TI refinement improves continuous position accuracy. Under the representative ablation settings, the complete method succeeds in all three sequences with a mean error of 1.95 m; the broader multi-start statistics are reported separately in Section 4.3.

## 5. Discussion

The results indicate that initialization performance mainly benefits from three linked operations: representing self-motion as ordered local route structures, retaining several plausible local correspondences, and confirming them through route-level topology. This combination is particularly useful in road networks with repeated local patterns, where several candidates can obtain similar local scores.

Different road-network patterns emphasize different aspects of the framework. Sequence 00 contains sparser normal-turn evidence and stronger regular-grid ambiguity, which is reflected by the lowest multi-start success rate (84.4%), the largest average required trajectory length (1.91 km), and the smallest successful-trial confirmation margin (14.48). Sequence 08 benefits strongly from internal direction-change augmentation and attains a 91.1% success rate with an average confirmation margin of 30.37. Sequence 09 contains richer turning-event evidence but still exhibits locally repeated structures, producing a 91.1% success rate and an average confirmation margin of 28.77. These differences are consistent with the role of event availability and chain-level discrimination in the proposed method.

The results also support the route-structure representation motivated by biological navigation. In insect route following, path integration provides self-motion information, while recognizable route cues and their sequence support familiar-route navigation [8,9,10,11,12,13,14,15]. In the proposed framework, odometry provides the self-motion basis, direction-change structures provide local route cues, and candidate-chain confirmation preserves their sequential topological relations. The single-turn-event and no-chain-confirmation ablations show that accumulating such evidence along the route improves initialization reliability.

The proposed method still has several applicability boundaries. First, candidate-chain confirmation requires sufficient normal turning events. The expanded multi-start experiment shows that starts in long-straight or sparse-event regions may require substantially longer observation distances before enough structure pairs are accumulated, and some such trials fail. Second, the external input is a standard road-network map, but the matching algorithm operates on an automatically enhanced internal representation. Severe road omissions, incorrect connections, or erroneous key topological relations in the input map can therefore prevent the correct candidate from being generated even after enhancement. Third, the method relies on absolute heading and local motion morphology from odometry; increased heading drift or shape distortion can affect both candidate retrieval and TI refinement. These conditions define the practical operating range of the current implementation.

The larger-map experiment shows that candidate retrieval and candidate-chain confirmation dominate the online runtime as the number of map candidates and competing chains increases. The average runtime rises from 3.28 s at 1 × 1 km to 29.80 s at 4 × 4 km. Future work will focus on hierarchical candidate indexing, parallel chain expansion, robustness to map and odometry errors, and confirmation under sparse-event conditions.

## 6. Conclusions

Motivated by the coordinated use of self-motion information, recognizable route cues, and route continuity in biological navigation, this paper proposes a topology-consistent trajectory-event matching method for GNSS-denied vehicle initial localization. By constructing trajectory structure pairs from adjacent normal turning events and generating corresponding candidates from an automatically enhanced standard road-network input, the method converts an initial odometry trajectory into a map-referenced starting position through local structural matching, candidate-chain confirmation, and translation-only local refinement.

With 45 systematically sampled starting positions in each of three KITTI-360 sequences, the method achieves an overall initialization success rate of 88.9%. Successful trials have a mean error of 2.46 m and a median error of 2.29 m, while the overall SR@5 m is 85.2%. These results show robustness to starting-position variation within the evaluated road scenarios. The lower success rate and longer required observation distance in sparse-event or locally ambiguous sections indicate that these conditions remain the principal limiting cases. Ablation results further demonstrate the importance of trajectory structure pairs, map-side internal direction-change augmentation, and candidate-chain confirmation.

The current method depends on sufficient normal turning events, reliable road-network topology, and stable absolute heading during initialization. Future work will therefore examine broader road networks, map and odometry perturbations, sparse-event scenarios, and more efficient candidate indexing.

## Figures and Tables

**Table 1:** Topology-consistent trajectory-event initial localization.

Step	Operation	Summary
Input	Load inputs	Standard road-network map in the coarse search region; odometry trajectory with absolute north-referenced heading.
1	Build structural representations	Convert the map to ENU and construct the enhanced directed topology; extract trajectory direction-change events and form ordered structure pairs from adjacent normal turns.
2	Retrieve local candidates	Generate map-side structure pairs; apply direction, length, turning, and morphology constraints; rank feasible candidates and retain Top-*N* hypotheses.
3	Confirm candidate chain	Connect topology-consistent candidates with limited one-step bridging, control expansion by beam search, and select a valid chain using chain-level confirmation criteria.
4	Refine and return position	Perform translation-only TI alignment around the first confirmed structure and return the continuous initial position; otherwise record failure or timeout.

**Table 2:** Main algorithmic parameters used in the experiments.

Module	Parameter	Value
Map topology	Bend-point deflection threshold	10°
Map topology	Internal-turn promotion threshold	17°
Map topology	Maximum road-segment interpolation length	30 m
Continuous-curvature edge	Mean curvature/cumulative turn/minimum length	0.15°/m/25°/10 m
Trajectory events	Active-interval on/off thresholds	0.4/0.3°/m
Trajectory events	Normal-turn/U-turn angle criterion	[25°, 155°)/≥155°
Trajectory structure pair	Minimum middle-corridor length	8 m
Local candidate filter	Absolute middle/entering/leaving direction errors	≤30°/≤42°/≤42°
Local candidate filter	Length ratio/absolute local-turn error/absolute corridor-turn error	≥0.38/≤65°/≤150°
Local candidate score	Weights (mid, pre, post, length, Δ_1_, Δ_2_)	0.30, 0.20, 0.20, 0.50, 0.06, 0.06
Candidate retrieval	Retained hypotheses	Top-*N* = 20
Candidate chain	Beam width	180
Candidate chain	Fault-tolerant bridge	Single-step bridge, *Z_i_* → *Z_i_*_+2_
Chain confirmation	Coverage/missing/uniqueness criteria	coverage ≥ 0.75; missing ≤ 1; score gap ≥ 1.5; score ratio ≥ 1.10
TI refinement	Rotation mode	No rotation
TI refinement	Arc-length search/fine step/grid	±25 m/0.25 m/0.5 m

**Table 3:** Contextual comparison of reported initialization errors on KITTI/KITTI-360 sequences.

Sequence	OpenStreet-SLAM/m	MPF/m	TOM-Odometry/m	Ours/m
00	2.24	~2.13	2.45	2.12
08	7.01	~6.12	6.70	1.91
09	3.49	4.83	4.45	1.82
Average	4.25	~4.36	4.53	1.95

Note: Results of OpenStreetSLAM, MPF, and TOM-Odometry are taken from [19]. Values marked with “~” are approximate. Because sensor inputs, map representations, and evaluation protocols differ among methods, the reported values are used as contextual references for the corresponding accuracy levels.

**Table 4:** Multi-start initialization statistics. Error statistics are computed over successful trials only.

Sequence	Starts	Success Rate	SR@5 m	Mean/m	Median/m	Std./m	Range/m
00	45	84.4%	80.0%	3.009	2.876	0.900	2.105–6.650
08	45	91.1%	88.9%	2.250	2.223	0.629	1.604–5.682
09	45	91.1%	86.7%	2.154	2.012	0.923	1.430–6.153
Overall	135	88.9%	85.2%	2.457	2.291	0.903	1.430–6.650

**Table 5:** Average map/search complexity and online runtime across the three sequences at representative map sizes.

Map Size	Road Segments	Enhanced Nodes	Map Candidates	Chain Expansions	Retr. (s)	Chain (s)	Total (s)
1 × 1 km	185	174	451	730	0.771	1.799	3.283
2 × 2 km	740	696	1804	1084	2.576	6.011	9.300
3 × 3 km	1666	1565	4060	1412	5.149	12.014	17.877
4 × 4 km	2962	2782	7218	1736	8.727	20.363	29.803

**Table 6:** Ablation outcomes. Numeric entries are localization errors for successful cases; failures and timeouts are shown explicitly.

Variant	Seq. 00	Seq. 08	Seq. 09	Successful Sequences
No internal points	4.33 m	Fail	Fail	1/3
Single-turn event	Timeout	2.88 m	Timeout	1/3
No chain confirmation	Fail	Fail	Fail	0/3
No TI refinement	4.33 m	9.46 m	9.51 m	3/3
Complete method	2.12 m	1.91 m	1.82 m	3/3

## Data Availability

The raw trajectory data used in this study are publicly available from the KITTI-360 dataset, and the road-network data were obtained from OpenStreetMap. The processed data supporting the reported results, including multi-start experimental results, computational scalability and ablation results, algorithm parameters, and representative intermediate outputs, are available on Zenodo at https://doi.org/10.5281/zenodo.23280576 (Version 1.0.0).
